# Elucidation of the Molecular Interaction Network Underlying Full-Length FUS Conformational Transitions and Its Phase Separation Using Atomistic Simulations

**DOI:** 10.1021/acs.jpcb.5c02911

**Published:** 2025-08-22

**Authors:** Shuo-Lin Weng, Priyesh Mohanty, Jeetain Mittal

**Affiliations:** † Department of Chemistry, 14736 Texas A&M University, College Station, Texas 77843, United States; ‡ Artie McFerrin Department of Chemical Engineering, Texas A&M University, College Station, Texas 77843, United States; § Interdisciplinary Graduate Program in Genetics and Genomics, Texas A&M University, College Station, Texas 77843, United States

## Abstract

Fused in Sarcoma (FUS) is a multidomain nucleic acid binding protein which orchestrates cellular functions such as gene expression, transcription, and DNA repair through liquid–liquid phase separation (LLPS). While crucial to understanding cellular processes, an atomic-level view of the molecular-level interactions associated with full-length (FL) FUS LLPS remains challenging due to its low solubility *in vitro*. Here, using all-atom (AA) molecular dynamics (MD) simulations, we examined the conformational dynamics and interactions of FL FUS in both dilute and condensed phases. Comparing two modern force fields (FFs)—Amber ff03ws and ff99SBws-STQ, we found that monomer simulation ensembles generated by both FFs exhibited qualitatively similar intramolecular interaction profiles dominated by intrinsically disordered regions (IDRs). While the two folded domains minimally participated in intramolecular interactions, their stabilities significantly influenced the chain dimension and led to discrepancies compared to experimental data for both FFs. We observed that the Amber ff99SBws-STQ coupled with parameters adopted from the Zinc Amber force field (ZAFF) maintained stable folded domains and improved estimates of the chain dimensions. Finally, a microsecond-time scale simulation of FL FUS condensate revealed an extensive network of electrostatic interactions which are strongly correlated with those that modulate the dilute phase conformations. Overall, insights from our AAMD simulations illuminate the interplay between folded domain stability and IDR interactions in modulating protein conformation and phase separation.

## Graphical abstract

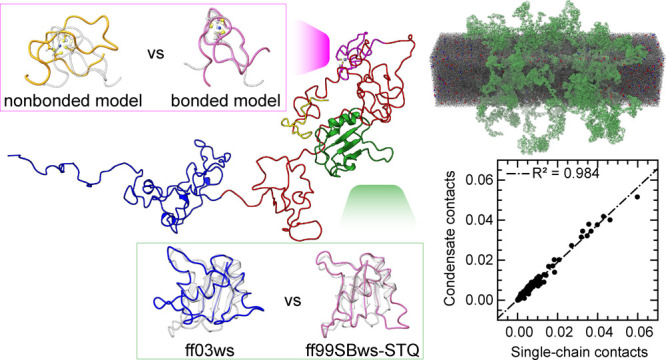

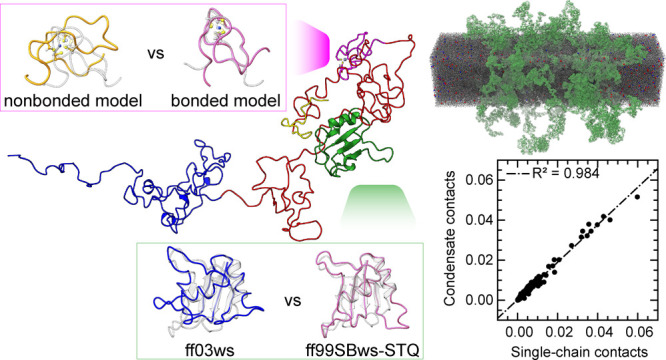

## Introduction

Fused in Sarcoma (FUS) is a multidomain RNA- and DNA-binding protein (526 amino acids) that comprises a disordered N-terminal low-complexity (LC) domain, two folded domains—an RNA recognition motif (RRM) and a zinc finger (ZnF) domain, three arginine–glycine–glycine-rich (RGG) domains, and a short nuclear localization sequence (NLS) (Figure 1).^1–3^ Collectively, RRM, ZnF, and RGG1–3 form the RNA-binding domains (RBDs). FUS has been demonstrated to undergo liquid–liquid phase separation (LLPS) *in vitro*,^4–8^ and participate in the formation of various membraneless organelles (MLOs) such as stress granules and nuclear bodies, contributing to cellular functions such as transcription, splicing, and DNA repair.^9–13^ Specifically, current studies indicate that FUS plays a critical role in several DNA damage response (DDR) pathways through its multivalency and ability to undergo LLPS, which are essential for initiating DDR and facilitating the recruitment of repair factors at damage sites.^14–18^ Moreover, FUS is also shown to interact with RNA polymerase II to regulate transcription and gene expression.^19–21^ Notably, FUS dysregulation has been associated with neurodegenerative diseases, such as Amyotrophic Lateral Sclerosis (ALS) and Frontotemporal Dementia (FTD) which are related to its cytoplasmic mislocalization,^22–24^ and in the development of cancers possibly due to impaired gene expression and DNA repair activity.^25–27^ These disease associations establish FUS as a key model protein for uncovering the molecular mechanisms underlying these pathologies.^5,28–32^

**Figure 1:**
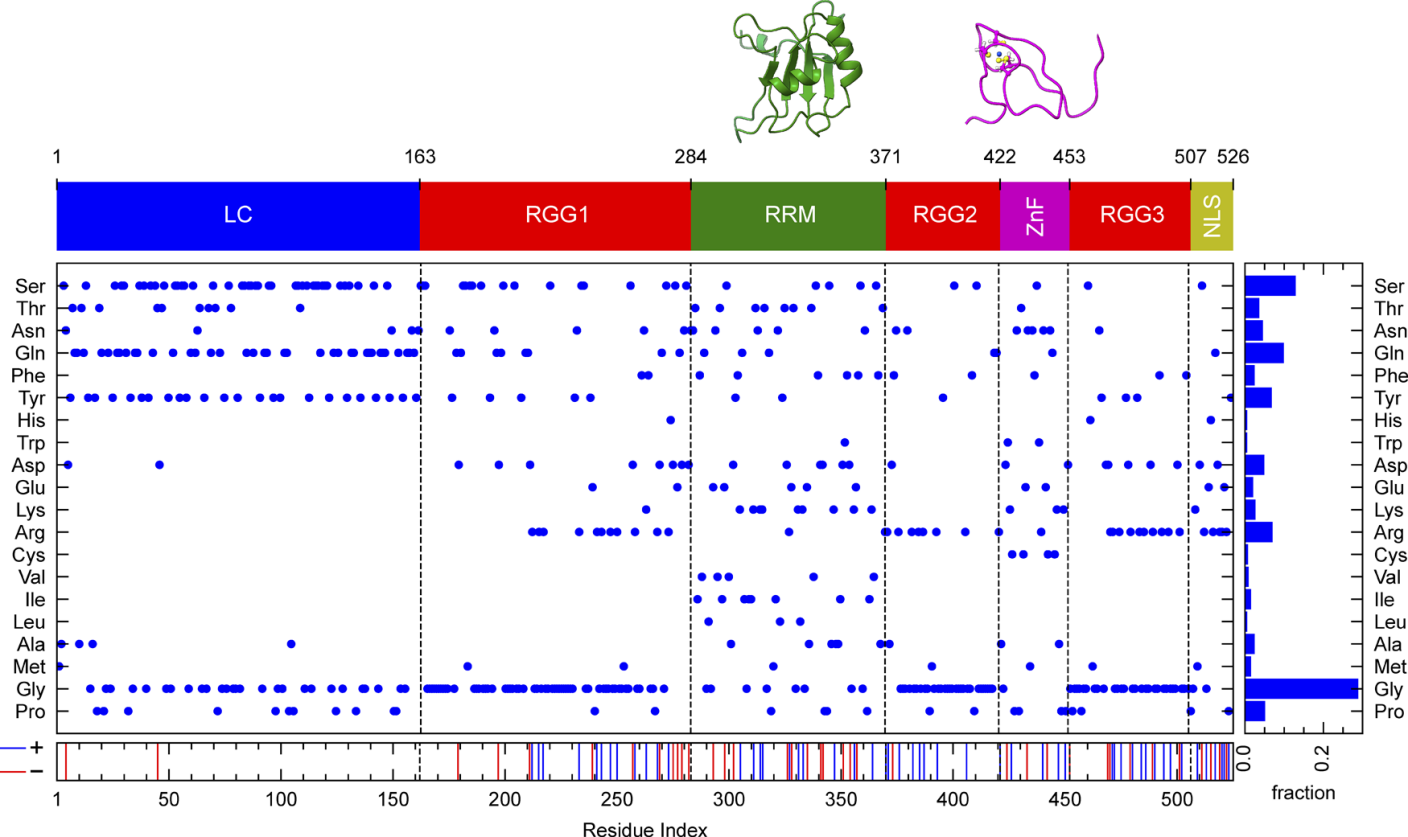
Domain structure and residue composition of FL FUS. FUS consists of a prion-like low-complexity domain (LC, aa 1–163), three arginine-glycine-glycine rich domains (RGG1, aa 164–284; RGG2, aa 372–422; RGG3, aa 453–507), two folded domains: the RNA-recognition motif-containing domain (RRM, aa 285–371) and the zinc-binding finger domain (ZnF, aa 423–453), and a nuclear localization sequence domain (NLS, aa 508–526). Residue composition is detailed for the FL sequence. The coloring scheme for each domain is consistent throughout the study. Structures of the folded domains are taken from the Protein Data Bank (PDB IDs: 6GBM and 6G99).

While the LC domain is capable of undergoing phase separation by itself,^7^ RBDs significantly enhance and modulate LLPS.^4,20,33,34^ Mutagenesis experiments, solution-state nuclear magnetic resonance (NMR) studies, and computational investigations have demonstrated the major driving forces of FUS LLPS, including the underlying residue-level interactions such as π-π, cation-π, hydrogen bonding, and electrostatic interactions.^7,20,33,35–39^ However, gaps remain in understanding the interplay between each domain in the context of full-length (FL) FUS, due to the challenges associated with its low solubility *in vitro*.^4,31^ Specifically, there are insufficient atomistic details about the various types of interdomain interactions in FL FUS and a limited understanding of the role of folded domains in phase separation. Interestingly, the RRM domain of FUS and other aggregation-prone proteins exhibits low thermal stability (*T*_m_ ∼ 50 °C), which may promote misfolding and self-assembly into amyloid fibrils, crucial for FUS cytotoxicity *in vivo.* ^40–42^ Stabilization of the RRM domain by the chaperone HspB8 can slow the aging process of FL FUS liquid droplets, suggesting that RRM stability impacts intracondensate dynamics.^43^ On the other hand, NMR experiments hint at transient interdomain interactions between the RRM and other domains,^40^ although atomistic details are lacking. The ZnF domain adopts a folded conformation when bound to a zinc ion, and its removal leads to unfolding.^34,44^ The presence of excess zinc ions in solution can improve the stability of ZnF, enhancing the LLPS of FUS, and promoting the cytoplasmic aggregation of FUS.^44,45^ Understanding the molecular mechanisms underlying RRM misfolding, particularly the influence of interdomain interactions on its conformational stability, and on the overall chain dimensions and phase separation of FUS, can provide crucial insights into disease mechanisms and the development of targeted therapeutics.

To address the above-specified knowledge gaps, we utilized all-atom (AA) explicit-solvent molecular dynamics (MD) simulations to investigate the interdomain and residue-level interactions of FUS in the dilute (monomeric) and condensed phases. MD simulations, complementing experimental approaches,^46,47^ have been successfully applied to understand LLPS behavior of intrinsically disordered regions (IDRs) and multidomain proteins such as TDP-43, hnRNPA1, hnRNPA2, NDDX4, and FUS.^7,20,35–39,48–55^ By leveraging simulations at varying model resolutions, these studies provided critical insights into conformational ensembles, molecular interaction networks, intracondensate dynamics, and the structural evolution of biomolecular condensates. In this study, we first performed AAMD simulations of the FL FUS monomer using two modern force fields (FFs)—Amber ff03ws and ff99SBws-STQ,^56–58^ and characterized the conformational ensembles in terms of their overall chain dimensions and the underlying intramolecular (both interdomain and residue-level) interactions. Notably, the ff03ws ensemble displayed significant chain compaction compared to ff99SBws-STQ which was associated with the partial unfolding of both folded domains. In contrast, our analysis indicated that ff99SBws-STQ stabilized RRM, and the incorporation of additional parameters adopted from the Zinc Amber force field (ZAFF)^59^ further maintained the stability of ZnF. With this modified FF (ff99SBws-STQ+ZAFF), we performed a large-scale simulation of the FL FUS condensate (comprising of 25 chains for 2.5 μs), which provided both a domain- and residue-level view of the interactions which stabilize the condensed phase. Further, our comparative analysis of the dilute and condensed phase contacts revealed a strong positive correlation for pairwise residue-level contacts formed in these phases, thereby enabling a logical extension of the established positive correlation between single-chain and condensed phase interactions of intrinsically disordered polypeptides (IDPs)^60–62^ to multidomain proteins. In conclusion, by elucidating how folded domains modulate FUS conformation through structural fluctuations in addition to residue-level IDR interactions which exhibit a strong positive correlation between dilute and condensed phases, our work advances a deeper mechanistic understanding of the FUS conformational landscape and its phase separation with important implications for its role in disease pathways.

## Methods

### All-Atom MD Simulations

#### Initial Structures and Molecular Modeling

The initial conformations for AA simulations of the FL FUS single chain were taken from the HPS-Urry coarse-grained (CG) simulation with a single bead per-residue resolution.^63,64^ Based on the AlphaFold2-predicted structure (https://alphafold.ebi.ac.uk/entry/P35637),^65,66^ RRM (residues 285–369) and ZnF (residues 422–453) domains were set as rigid bodies, while the rest of the protein was kept flexible. The CG simulation was performed at 300 K using the LAMMPS software package.^67^ Random conformations were sampled over a 1 μs CG simulation and converted to AA configurations using the MODELLER package,^68^ using solution-state NMR structures (PDB IDs: 6GBM and 6G99 ^69^) as templates for the folded domains.

For isolated folded domain simulations, protein structures were taken directly from the corresponding NMR structures, excluding the RNA (PDB IDs: 6GBM and 6G99 ^69^).

To model the FL FUS condensate, a pre-equilibrated configuration with 25 protein chains was extracted from a CG phase coexistence simulation conducted at 300 K using HOOMD-Blue 4.7.0.^70^ The MODELLER package was used to reconstruct the all-atom slab configuration, again incorporating NMR structures as templates for the folded domains.^69^ The subsequent AA simulation, described below, refines local interactions and provides atomistic detail within this physically relevant framework. This multiscale approach followed established protocols from our previous studies.^33,35,48^

#### Force Field Choice and Simulation Protocol

All systems were simulated in explicit solvent, modeled using the Amber ff03ws or ff99SBws-STQ force fields and the TIP4P/2005 water model,^56–58,71^ both incorporating improved NaCl parameters.^72^ Zinc ion binding within the ZnF domain was represented using two parameter sets: nonbonded (NBM) and bonded (ZBM) models. The NBM approach utilized CYZ parameters as outlined by Macchiagodena et al.,^73^ which we validated in a prior study examining zinc-mediated modulation of SOD1 protein conformations.^74^ The ZBM approach employed the Zinc Amber Force Field (ZAFF) developed by Peters et al.^59^ The protein molecules were placed in boxes of appropriate dimensions: an octahedron (edge length: 15 nm) for FL FUS single-chain simulations, a cubic box (edge length: 6 nm) for isolated folded domains, and a 12.5 × 12.5 × 50 nm^2^ tetragonal box for the FL FUS slab. The systems were solvated with counterions added to achieve electroneutrality and a salt concentration of 150 mM to mimic physiological conditions.

Each system underwent energy minimization with the steepest descent algorithm, followed by a 100 ps NVT equilibration at 300 K. For the condensate simulation, an initial 250 ps annealing cycle (5–300 K) with position restraints applied to backbone heavy atoms preceded the NVT equilibration. The velocity rescaling algorithm was used for temperature control with a coupling constant of 0.1 ps.^75^ Subsequently, a 100 ps NPT equilibration was conducted using the Parrinello–Rahman barostat with a coupling constant of 2 ps for pressure control at 1 bar.^76^ These initial equilibration simulations were performed using the classical MD package GROMACS-2022,^77,78^ employing periodic boundary conditions and a 2 fs integration time step.

Production simulations were performed using Amber 22 for its high performance in standard production runs,^79^ and OpenMM 8.1.1 for its flexibility in applying custom restraints.^80^ For Amber simulations, the structure and topology files obtained from GROMACS were converted using the ParmEd package in AmberTools 23, while OpenMM simulations directly utilized the GROMACS files. Hydrogen mass repartitioning was applied in both softwares to enable a 4 fs time step.^81^ All production simulations were performed in the *canonical ensemble*, with Langevin dynamics^82^ controlling the temperature at 300 K (friction coefficient = 1 ps^–1^). Short-range nonbonded interactions were calculated with a 0.9 nm cutoff, and long-range electrostatic interactions were computed using the particle mesh Ewald (PME) algorithm.^83^ Bond constraints for hydrogen-containing bonds were enforced in OpenMM and Amber with the SHAKE algorithm.^84^ For the RRM domain, restraints to maintain the native folded state were implemented in OpenMM using the CustomBondForce class to apply a flat-bottom restraint potential based on NMR-derived hydrogen-bond information. The potential function is

$$V\left(r_{\mathrm{ij}}\right)=\{\begin{array}{l} \frac{1}{2}k{\left(r_{\mathrm{ij}}-r_{0}\right)}^{2},,r_{\mathrm{ij}}<r_{0}\\ 0,,r_{0}<r_{\mathrm{ij}}<r_{1}\\ \frac{1}{2}k{\left(r_{\mathrm{ij}}-r_{1}\right)}^{2},,r_{0}<r_{\mathrm{ij}}<r_{1}\\ \frac{1}{2}k\left(r_{2}-r_{1}\right)\left(2r_{\mathrm{ij}}-r_{2}-r_{1}\right),,r_{2}<r_{\mathrm{ij}} \end{array}$$

where *k* = 20 kcal/nm^2^, *r*_0_ = 0.27 nm, *r*_1_ = 0.3 nm, and *r*_2_ = 0.35 nm. Aside from the additional custom restraints applied in OpenMM, both engines used identical parameters, making their results directly comparable.

After production runs, the Amber NetCDF or CHARMM DCD trajectory files were converted to GROMACS compressed trajectory files *via* the CPPTRAJ package^85^ in AmberTools 23 for further analysis. A concise summary of the single-chain simulations is provided in Supporting Table 1. The system size of the all-atom slab simulation is described in Supporting Table 2.

### MD Trajectory Analysis

Protein–protein pairwise contacts were calculated using MDAnalysis 2.5.0,^86,87^ following the methodology defined in our previous work.^33^ A contact was considered formed if any heavy atoms from two residues were within 4.5 Å. Residue pairwise contacts were determined by summing all contacts between heavy atom pairs from the respective residues, excluding interactions involving residues within a five-residue proximity. We note that summing all heavy-atom contacts for all residue-type pairs may introduce a bias toward larger, planar residues, potentially underestimating the contributions of smaller or linear side chains. However, since these larger residues are expected to contribute more favorably toward pairwise residue-level interactions (*via* additional atomic contacts), we believe this definition adequately describes the true amino acid-type interaction propensities. Moreover, this definition can effectively distinguish strong pairwise interactions from weaker ones, unlike the traditional binary contact definition.

For the angle–distance distribution analysis between phenylalanine and arginine residues (F-R contacts), the angle (θ) is defined between the vectors perpendicular to the guanidinium plane of arginine and the phenyl ring of phenylalanine. The distance (*r*) is measured as the separation between the centers of mass of their heavy atoms. These definitions follow protocols established in our previous studies.^33,88^ The resulting distribution was normalized according to a geometric correction scheme similar to that of Marsili et al.^89^ The pair correlation function is defined as

$$g_{\mathrm{FR}}\left(r,\theta \right)=\frac{\sum_{i}^{N_{F}}\sum_{j}^{N_{R}}\delta \left(r-r_{\mathrm{ij}}^{\mathrm{FR}}\right)\delta \left(\theta -\theta_{\mathrm{ij}}^{\mathrm{FR}}\right)}{r^{2}\sin\theta }$$

where δ is the Dirac delta function.

The *gmx polystat* command was used to compute the radius of gyration (*R*_g_) and end-to-end distance (*D*_ee_), while their corresponding autocorrelation functions were calculated with the *gmx analyze* command with “*-ac*” flag. The hydrodynamic radius (*R*_h_) was calculated using our custom script implementing the HullRad algorithm^90^ with MDAnalysis,^86,87^ enabling trajectory-wide analysis with multiprocessing. The *gmx sasa* command was used to do Solvent Accessible Surface Area (SASA) analysis. Root-mean-square deviations (RMSD) and fluctuations (RMSF) for the folded domains were calculated with CPPTRAJ.^85^ Visualization of snapshots was performed using ChimeraX.^91^

The first 1 μs of each single-chain simulation was skipped as equilibration, based on the chain relaxation time estimated from the *R*_g_ autocorrelation functions (Figure S1A,B). Density profiles of the condensed phase were generated from the final 200 ns of simulation trajectories using the *gmx density*. Local ion concentrations were predicted using a simple model proposed in prior studies.^35^ To assess convergence of the dense phase, we performed time-series analyses of per-chain radius of gyration (*R*_g_), end-to-end distance (*D*_ee_), root-mean-square deviation (RMSD), and total contact counts (Figures S6A,B and S7A). As indicated by total contact (Figure S7A), the first 1 μs of the slab simulation was also treated as equilibration.

Except for the isolated ZnF simulation with ZAFF, all statistical data from single-chain simulations averaged over three independent trajectories. For the condensed phase simulation, all data presented in this study averaged over 25 chains.

## Results

### Conformational Ensembles of FL FUS Monomers Reveal Force Field-Dependent Chain Dimensions and Similar Interaction Networks

We chose two state-of-the-art force fields to model the FL FUS monomer ensemble. Our initial choice—ff03ws,^56,57^ was motivated based on consistency with previous studies on FUS fragments.^7,33,35^ However, in simulations of the full-length protein, we observed that while ff03ws performs well for IDRs,^92^ it was unable to maintain the stability of both folded domains—a point we elaborate on later. To overcome this limitation, we utilized the ff99SBws–STQ force field,^58^ which we employed in our recent study to investigate the conformational ensemble of full-length TDP-43.^48^ This force field has proven effective at preserving the native structure of folded domains while accurately capturing IDR behavior.^88,93,94^

The radius of gyration (*R*_g_) and hydrodynamic radius (*R*_h_) are widely used metrics to characterize the overall chain dimensions and structural flexibility of multidomain proteins such as FUS, as most regions are disordered.^95^ Our simulations show distinct distributions of *R*_g_ between the two force fields, reflecting their differences in terms of the sampled conformations (Figure 2A,B). The ff03ws model produced a collapsed and less flexible ensemble with a mean *R*_g_ of 2.6 ± 0.1 nm. In contrast, the ff99SBws-STQ model exhibited a more expanded conformational ensemble with a mean *R*_g_ of 5.0 ± 0.9 nm. Our results are similar to those recently reported by Sarthak et al., in which ff03ws gave rise to a collapsed ensemble, whereas ff99SB-disp yielded expanded conformations—both under simulation conditions of 150 mM KCl.^96^ Sarthak et al. also reported an experimental *R*_g_ distribution ranging from 3–5 nm, measured by dynamic light scattering (DLS) experiments in the presence of 1 M KCl.^96^ However, such high salt concentrations are likely to impact the conformational ensemble of FUS due to charge screening or salting-out effects.^33,97^ Likewise, Yoshizawa et al. reported an expanded conformational ensemble (*R*_g_ = 4.87 nm) for maltose binding protein (MBP)-tagged FUS in the presence of 0.5 M NaCl from small-angle X-ray scattering (SAXS) experiments.^98^

**Figure 2:**
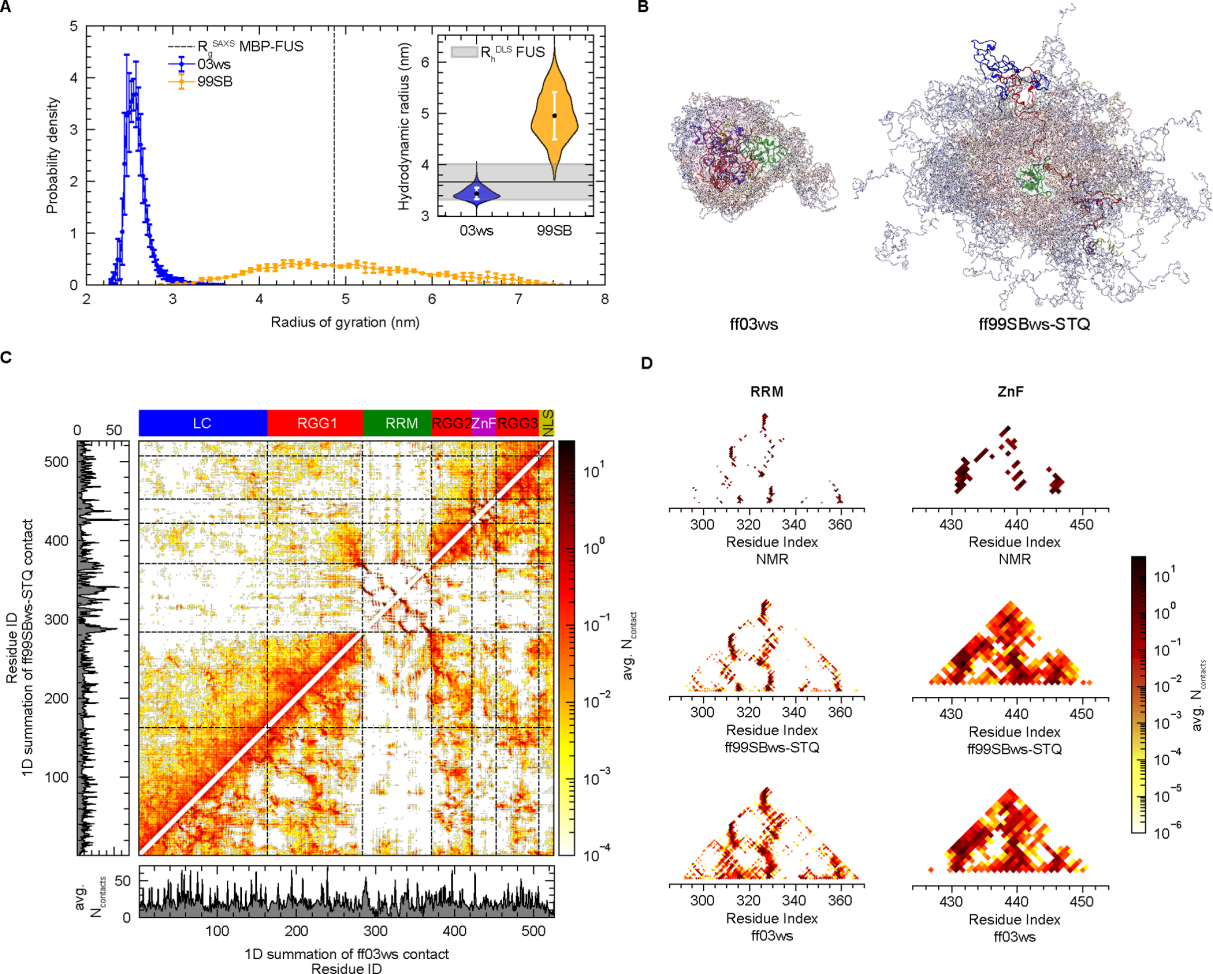
Conformational ensembles and interaction profiles of the FL FUS monomer. (A) Distribution of the radius of gyration (*R*_g_) from atomistic simulations using ff03ws and ff99SBws-STQ force fields, along with the experimental MBP-FUS value.^98^ Inset: Violin plot of hydrodynamic radius (*R*_h_) distributions from both models and the experimental value.^17^ (B) Snapshots of conformational ensembles of FL FUS. (C) Intramolecular contact profiles showing heavy atom contacts for each position, averaged over simulation ensembles for the given period. See Methods section for the definition of contact. The left column and bottom row represent one-dimensional summations. The top color bar indicates domain ranges. (D) Contact profiles of the folded domains (RRM and ZnF) from simulation and NMR ensembles.^69^

At 200 mM NaCl, Sukhanova et al. reported the average *R*_h_ of FUS from DLS experiments as 3.67 ± 0.35 nm.^17^ For comparison, the *R*_h_ calculated from the ff03ws simulations using the HullRad algorithm^90^ was 3.44 ± 0.12 nm, while the ff99SBws-STQ simulations yielded an *R*_h_ of 4.96 ± 0.46 nm (Figure 2A). These results indicate that the mean *R*_h_ of FL FUS lies much closer to ff03ws although the overall chain dimension is still underestimated by ∼7%. Overall, while each model captures aspects of the experimental conformations, neither fully encompasses the experimentally observed native state ensemble, reflecting the inherent limitations of these force fields in fully reproducing FUS’s conformational diversity and challenges associated with experimental determination of *R*_g_ or *R*_h_.

To explore the relationship between the observed chain dimensions and interdomain interactions in the FL FUS conformational ensembles, we computed the intramolecular contacts for both force fields (Figure 2C). Previous studies of IDPs suggest that the intramolecular interactions governing single-chain conformations in the dilute phase largely resemble those promoting LLPS for low complexity IDR sequences such as those occurring in FUS.^33,50,60–62,88^ Additionally, for multidomain proteins, specific intra- and interdomain interactions also affect their propensity to undergo LLPS.^48,99,100^ Interestingly, despite the quantitative differences in terms of overall contact counts due to distinct conformational preferences, both models identify the similar primary interaction hotspots (Figure 2C). Specifically, extensive contacts were observed between the IDRs, consistent with previous studies.^20,33,34,40^ In contrast, only a few interdomain contacts were observed between the folded domains and the IDRs. Solvent accessible surface area (SASA) analysis indicates that the limited contact involvement of the RRM domain is likely due to its low surface accessibility (Figure S1C), while for the ZnF domain, the minimal contribution is primarily attributable to its short chain length. Interestingly, upon closer inspection of the contact maps for the folded domains (Figure 2D), we observed both RRM and ZnF domains in the ff03ws ensemble displayed a significant disruption of native pairwise contacts compared to the NMR structures,^69^ along with the emergence of numerous non-native contacts. In case of ff99SBws-STQ, while the RRM domain appears to maintain its native contact profile, non-native contacts were observed in the ZnF domain.

In conclusion, while our simulations revealed distinct conformational ensembles for FL FUS monomers across the two force fields, both consistently exhibited extensive contacts within IDRs and limited contributions from folded domains, highlighting the central role of IDR interactions in driving LLPS.^7,20,33^ Next, given the significant differences in contact formation across the two force fields for both folded domains, we aimed to examine how the structural integrity of these domains influence the overall conformational ensemble of the full-length protein. To this end, we examined their structural stabilities in the FL FUS construct and compared them to those of their isolated counterparts.

### Folded Domain Stability Modulates the Conformational Ensemble of FL FUS Monomer

While *in vivo* experiments have linked the stability of the RRM domain to the aging of FUS droplets, the molecular details and mechanisms remain unclear.^34,40,43,44^ The RBD segment cannot phase separate on its own but can undergo LLPS when interacting with nucleic acids such as RNA, DNA, and ATP, and has been shown to enhance the LLPS of the LC domain.^2,20,34,101^ However, prior studies often included contributions from other domains, such as the RGG regions, leaving the specific role of folded domains insufficiently understood. As stated previously, comparisons with native NMR structures reveal significant disruption of intradomain contacts in the RRM/ZnF domain with ff03ws and the ZnF domain with ff99SBws-STQ (Figure 2D), implying destabilization of these domains in both force field ensembles. To further assess the stability of folded domains within the FL FUS ensembles, we computed the root-mean-square deviation (RMSD) and root-mean-square fluctuation (RMSF) of their C_α_ atoms, using the experimental structures as references (PDB IDs: 6GBM for RRM, 6G99 for ZnF).^69^ We also compared these results to those of isolated folded domain simulations to distinguish the influence of the force fields from domain interactions within FL FUS.

The RRM domain of FUS shares a common structure with RNA-binding proteins like TDP-43, hnRNPs, and the FET family, featuring a domain architecture with a central β-sheet flanked by two α-helices (Figure 1).^69,102^ This domain has been shown to spontaneously self-assemble into cross-β-rich amyloid fibrils.^40^ The FL droplet with chaperone-stabilized RRM or RRM deletion displayed a delayed aging process.^43^ Additionally, structural comparisons between the isolated and the RNA-bound RRM indicate that RNA binding does not impact the RRM structure significantly.^69,102^ The ff03ws ensemble was found to populate high RMSD values exceeding 4 Å for RRM, while the ff99SBws-STQ ensemble exhibited a relatively stable RRM (Figures 3A,B and S2A). Similar to our simulations, Sarthak et al. also reported that the ff03ws ensemble was characterized by an unstable RRM domain.^96^ Overall, these observations are also consistent with previous reports of folded state destabilization for ff03ws,^103,104^ which likely arises due to an overestimation of protein–water interactions. A recently proposed variant of this force field—ff03w-sc—which selectively scales protein–water interactions for side chain atoms only (as opposed to both side chain and backbone atoms in ff03ws)—can be used to greatly improve the structural stability of folded domains.^94^ Interestingly, RRM stability was lower in the FL context compared to the isolated form for both force fields (Figure 3A). RMSF analysis indicated that the increase in RMSD in the full-length protein was associated with a global increase in the conformational flexibility of the domain backbone for both force fields (Figure S2D). Overall, the difference in stability between full-length and isolated domains suggests that the presence of disordered domains in the context of FL FUS destabilizes the RRM domain and increases its conformational flexibility.

**Figure 3:**
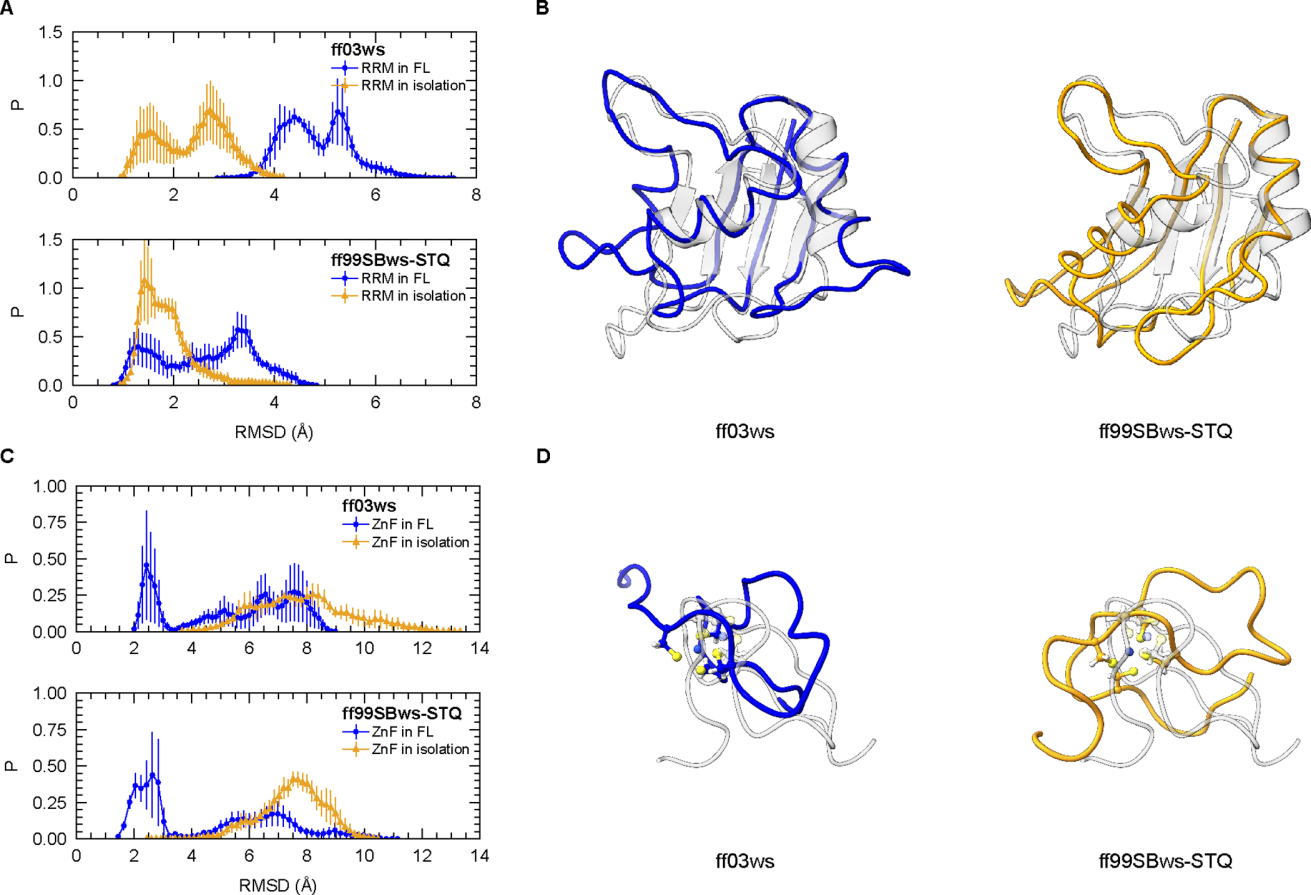
Comparison of folded domain stabilities between isolated and FL FUS constructs. (A) Distribution of average root-mean-square deviation (RMSD) of Cα atoms in the RRM domain (residues 285–369) from three independent replicas for each condition. RMSD is calculated relative to the reference PDB structure (see Figure 1). (B) Structure alignments to the NMR structure (PDB 6GBM, gray). The structures represent the final frame from one trajectory of each force field. (C) Distribution of average RMSD of Cα atoms in the ZnF domain (residues 423–453) from three independent replicas for each condition, relative to the reference PDB structure (see Figure 1). (D) Structure alignments to the NMR structure (PDB 6G99, gray). The structures represent the final frame from one trajectory of each force field.

After investigating the RRM domain, we then examined the ZnF domain, which is part of the RanBP2-type ZnF family and features a typical four-cysteine motif that binds to zinc. When a zinc ion and single-stranded RNA containing the GGU motif are present, the ZnF domain forms a structure with two crossed β-hairpins (Figure 1).^69^ The removal of zinc causes the ZnF domain to unfold (Figure S2C), but this unfolding is reversible upon reintroducing zinc ions.^34^ In order to maintain stable coordination of zinc to the cysteine tetrad in ZnF, we utilized nonbonded Zn-Cys interaction parameters (nonbonded model, NBM).^73^ The simulations with NBM showed partial to complete unfolding of ZnF in both force fields with the presence of zinc, whether in isolation or in the FL context (Figures 3C,D and S2B). Interestingly, unlike RRM, the ZnF domain is more stable in the FL context compared to the isolated form (Figures 3C and S2E), suggesting that the presence of other domains might enhance the conformational rigidity of ZnF. As with RRM, the ff99SBws-STQ model exhibits overall higher stability than ff03ws. Importantly, zinc remained bound to the cysteine residues, with small zinc–sulfur distances (Figure S3A) for nearly all trajectories. Only one trajectory for isolated ZnF showed the dissociation of Zn from one cysteine, occurring after unfolding (Figure S3B,C). Therefore, ZnF unfolding was not driven by the complete dissociation of zinc from ZnF. In conclusion, these unfolding results suggest the inability of nonbonded Zn-Cys interaction parameters to fully maintain the ZnF folded state, and stronger zinc-binding *via* covalent bonds might be critical to preserve the ZnF native structure. Therefore, we tested the ability of Zn-Cys bonded interaction parameters to maintain the native structure in the next section.

### Stabilization of Folded Domains Leads to Convergence of Single-Chain MD Ensembles between Both Force Fields

To better understand the influence of folded domain stability on the overall conformation of FL FUS, we implemented specific modifications aimed at improving domain folding. For the RRM domain in the ff03ws model, we applied additional distance restraints derived from NMR structures to maintain its secondary structure (see Methods section), effectively limiting its RMSD to less than 3 Å (Figure S4A). These restraints resulted in a significantly expanded overall protein conformation compared to the original ff03ws model (Figure 4A). This expansion likely arises because residues previously exposed in the unstable RRM, which interacted with other domains and promoted collapsed conformations, are no longer accessible. As anticipated, the structural restraints altered the interaction profile of RRM, reducing its interdomain interactions and concentrating them on specific surface-exposed residues (Figure S4B). Additionally, RRM contacts between the LC and RGG2/3 domains were notably reduced, due to an overall decrease in the number of RRM residues available for interdomain interactions. Collectively, these findings demonstrate that stabilizing the RRM domain affects the overall conformation of FL FUS while reducing its interdomain interactions with other IDR regions.

**Figure 4:**
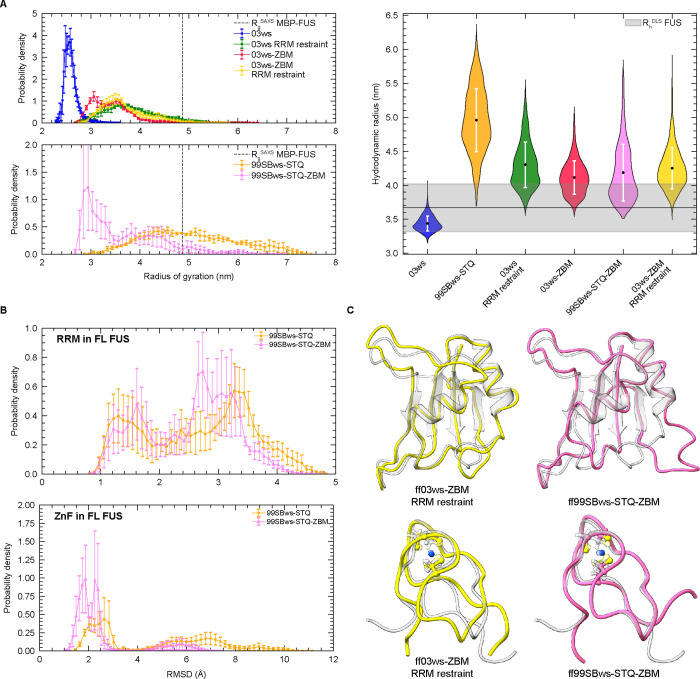
Increased stability of folded domains alters the chain dimensions of the FUS single-chain ensemble. (A) Left: Distribution of *R*_g_ from atomistic simulations of FL FUS with different force fields, along with the experimental MBP-FUS value.^98^ Right: Violin plot of hydrodynamic radius (*R*_h_) distributions for each force field and the experimental value.^17^ (B) Distributions of average RMSD of Cα atoms in the folded domains from two ff99SBws-STQ force fields. (C) Structure alignments to the NMR structures (PDB 6GBM and 6G99, gray). The structures represent the final frame from one trajectory of each force field.

To improve the folding of the ZnF domain, we incorporated the well-established Zn-Cys bonded parameters from the Zinc Amber force field (ZAFF) (zinc bonded models, ZBMs).^59^ The RMSD analysis indicated that the ZAFF parameters significantly improve the stability of ZnF native structure in both force fields, with RMSD values less than 3 Å for both FL FUS and the isolated domain constructs (Figure S4C). These results are in line with the observations made by Sarthak et al. wherein force fields using covalent bonds to describe Zn-Cys interactions showed higher ZnF stability.^96^ Analysis of Zn–S distances showed that the ZAFF parameters resulted in longer Zn–S bond lengths compared to the NBM, indicating that stabilization arises not from tight binding but from improved structural constraints (Figure S4D). Additionally, Zn–S–C_β_ angle analysis revealed a narrower range of distribution for ZAFF, implying that angle constraints contributed to improved ZnF folding (Figure S4E). However, these bond lengths remained longer than those derived from the NMR structure,^69^ and the angles of the four cysteines were the same in simulations, while the angles from the NMR structure varied, suggesting that further refinement of ZAFF may be required to improve the overall agreement with experimental results.

Interestingly, incorporation of the ZAFF parameters caused compaction of the protein chain for the ff99SBws-STQ ensemble, while chain expansion was observed for the ff03ws ensemble. Applying both RRM restraints and incorporating ZAFF parameters in the ff03ws model resulted in a further expanded conformation (Figure 4A). Despite deviations in chain dimensions from experimental data—likely due to the addition of tags, buffer conditions, and salt concentrations—the ff99SBws-STQ with ZAFF yielded acceptable RRM stability and significantly improved ZnF folding without the need of additional structural restraints (Figure 4B,C). On the other hand, compared to the ensemble obtained for the NBMs, the interaction profiles are similar, displaying dominant LC and RGG interactions with limited interdomain interactions involving folded domains. In the ZnF region, contacts are more concentrated and interdomain contacts are reduced, similar to the difference in the RRM region between ff03ws with and without RRM restraints (Figure S4B). Overall, these results highlight that while folded domains in their native state contribute minimally to direct interactions within FL FUS, their unfolding can profoundly impact the conformational ensemble and intramolecular interaction profile. These simulations underscore the importance of accurate modeling for folded domains in understanding the conformational properties of FUS and other multidomain proteins.

Interestingly, a slight increase in linear correlation emerged between the pairwise summations of contacts between both force fields when structural restraints are applied to stabilize folded domains (Figures 5A and S5). This indicates that contact-prone residue pairs are similar across both modified force fields. Arginine, tyrosine, glutamine, serine, and glycine residues emerging as dominant contributors to the intramolecular contacts across all simulations which is somewhat unsurprising, given that these are the five most abundant residue types in FL FUS (Figure 5B). Beyond the traditionally recognized major contributors to LLPS—tyrosine-tyrosine and arginine-tyrosine pairs—significant pairwise contributions from polar residues like glutamine and serine were observed.^4,33,88^ Notably, additional pairs involving arginine with other residues, particularly arginine-arginine interactions *via* π–π stacking, were observed, which is consistent with previous NMR and MD studies.^33^

**Figure 5:**
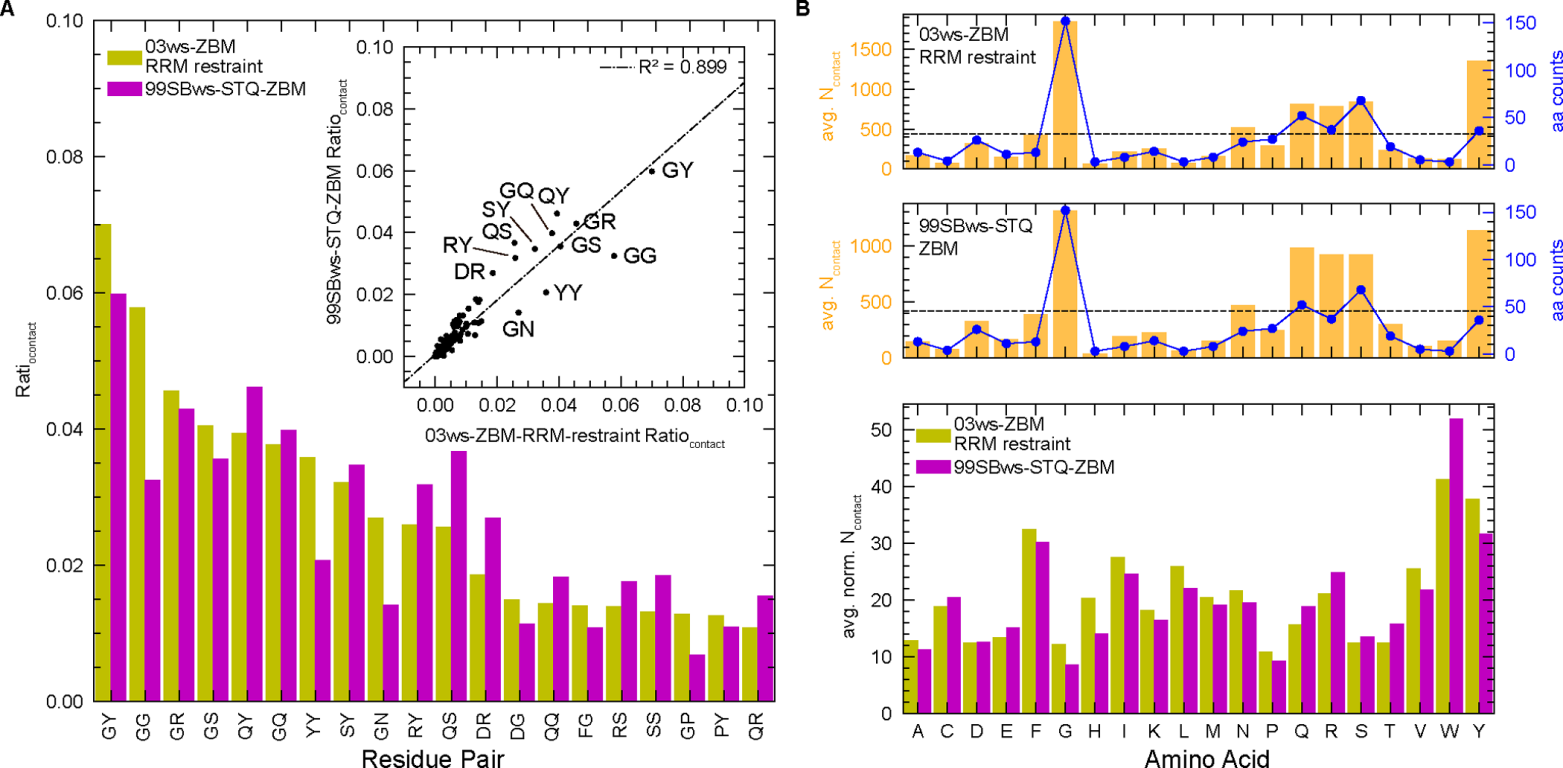
Residue-type interaction pairs modulating the monomeric conformational ensemble of FL FUS. (A) The top 20 residue-pair contacts from FL FUS single-chain simulations with conformational restraints applied to RRM/ZnF domains are shown. See Figure S5 for full rank. Contact ratios were calculated by dividing the number of individual contacts by the total number of contacts. Inset: Correlation between pairwise contact ratios in the ff03ws-ZBM RRM restraint (*x*-axis) and ff99SBws-STQ-ZBM (*y*-axis) models. (B) Top: Average number of contacts per residue type and corresponding amino acid counts (aa counts). Bottom: Normalized contact numbers for each residue type, calculated by dividing the number of contacts per residue type by its amino acid count.

### FL FUS Condensate Simulation Reveals an Elaborate Network of Electrostatic Interactions with Enhanced Folded Domain Stability

After investigating the interplay at the single-chain level, we aimed to characterize the network of intermolecular interactions and stabilities of the folded domain within the crowded environment of the condensate. To this end, we performed a large-scale simulation of the FL FUS condensed phase using a slab configuration containing 25 protein chains (Figure 6A), starting from an equilibrated state of the coarse-grained (CG) phase coexistence simulation. Given its ability to maintain the native structure of RRM without the need for additional structural restraints (Figure 4B,C), the ff99SBws-STQ with additional ZAFF parameters to further stabilize ZnF (ff99SBws-STQ-ZBM), was used to simulate the FL FUS condensate. With the availability of high-resolution atomistic simulation data, we examined water and ion partitioning within the condensed phase (Figure 6B). The FL FUS condensate protein concentration was approximately 500 mg/mL, with a water content of about 600 mg/mL, similar to previous FUS LC condensate simulations.^35,39^ Due to net positive charge of the FUS FL sequence, Cl^–^ ions exhibited a higher concentration inside the condensate compared to Na^+^ ions to maintain electroneutrality.

**Figure 6:**
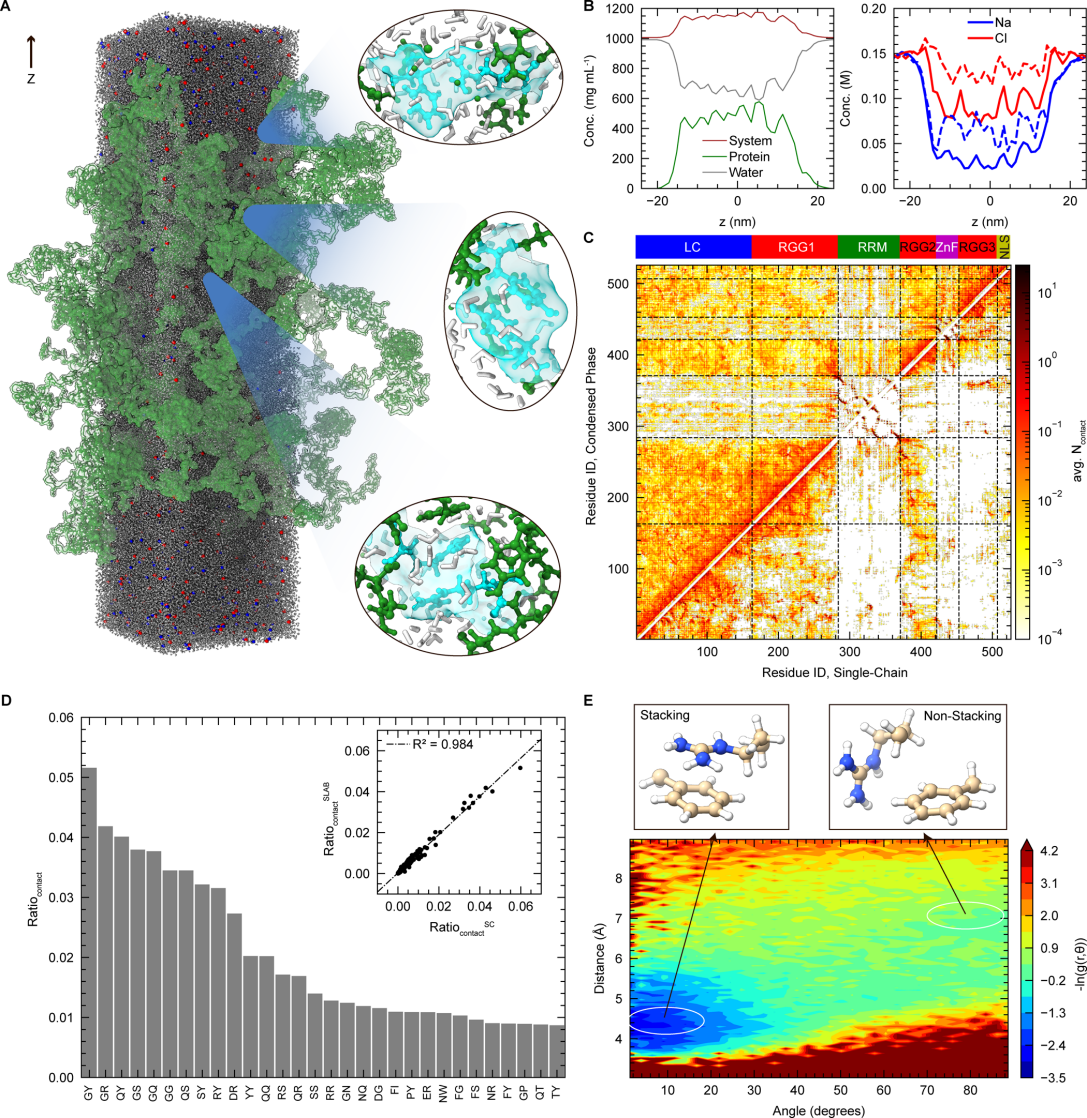
Single-chain interactions mirror those in FL FUS condensates. (A) Atomistic condensed phase simulation of 25 chains of FL FUS (green), with water (gray), Na^+^ (blue), and Cl^–^ (red). Insets display representative pairwise interactions—top: Phe–Arg, middle: Tyr–Tyr, bottom: Arg–Arg—along with surrounding atoms. Highlighted residues are shown in cyan, other residues in green, and water molecules in white. (B) Density profiles from atomistic FL FUS condensed phase simulation, along the *z*-axis shown in **6A**. Components are shown in the legend. Dashed lines in right indicate the predicted ion concentrations by using concentrations of protein cationic and anionic residues. See Methods section for details. (C) Contact profiles comparing FL FUS single-chain and slab simulations using ff99SBws-STQ-ZBM force field. Single-chain data averaged from three independent trajectories; slab data averaged across 25 chains. (D) Top 20 residue pairs with highest contact frequencies in FL FUS slab simulation. See Supporting Figure 7 for full rank. Inset: Correlation of pairwise contact ratios between single-chain (*x*-axis) and slab (*y*-axis) simulations. (E) Top: Schematic representation of pairwise F–R interaction types (stacking/nonstacking), analyzed in the FL FUS slab simulation. Bottom: Contour plot of the F–R pair correlation function. See Methods section for the definition of *g*(*r*,θ). Angle bins are 2.5°, and distance bins are 0.6 Å.

Comprehensive pairwise contact mapping revealed that (Figures 6C,D and S7C), tyrosine, arginine, glutamine, serine, and glycine form dominant contacts—consistent with their established roles in IDP phase separation and mirroring observations in FUS LC-RGG1 and other IDP condensates.^4,33,88^ Electrostatic interactions substantially contribute to the FL FUS interaction network due to abundant charged residues (Figure 1),^4^ as reflected in the high frequency of DR and ER contacts (Figure 6D). In contrast, residues within folded domains primarily formed stable intramolecular contacts and showed limited intermolecular interactions (Figure 6C), relative to the IDRs, in agreement with our single-chain simulation results. Notably, we identified previously hypothesized but unverified phenylalanine-arginine (F-R) interactions in FUS involving π-π stacking, confirmed through sp^2^/π plane angle-distance distribution analysis (Figure 6E).^33,89,105–107^ This multimodal interaction landscape—combining stereospecific strong contacts with abundant weak interactions—demonstrates how diverse physicochemical forces synergistically mediate phase separation in multidomain systems.^33,88^

Consistent with observations in FUS LC, LC-RGG1, and other IDP condensates, the protein chains expanded in the condensed phase.^33,35,108^ The distribution of *R*_g_ and D_ee_ shifted toward larger values in the slab simulation compared to the single-chain simulations (Figure S6C), resulting from a shift from intramolecular to intermolecular interactions within the condensate. Importantly, both RRM and ZnF domains remained folded for all chains throughout the trajectory (Figure S6B,D), indicating that the condensate environment did not significantly destabilize these structured regions. Previous studies on IDPs have highlighted the relationship between single-chain properties and condensate behaviors, suggesting that intramolecular interactions observed in the dilute phase can contribute to condensate stability.^33,35,48,60–62,99,109^ Analysis of the contact maps within the FL FUS condensate revealed similarities and differences compared to the single-chain profile (Figure 6C). As expected, interdomain contacts of the folded domains increased within the condensate due to enhanced opportunities for intermolecular interactions. Despite this, the overall interaction profile—characterized by the dominant role of IDR contacts and limited contributions from folded domains—remained remarkably consistent with the single-chain ensemble. This similarity is further supported by the high correlations observed in the per-residue and pairwise contact profiles (Figures 6D, and S7B,C), suggesting that the interactions governing monomeric conformations also play a crucial role in promoting and stabilizing LLPS,^60–62^ not only in IDRs but also in multidomain proteins.

## Discussion and Conclusions

FUS is a pivotal protein in various cellular functions due to its multivalency and capability to undergo LLPS. Understanding the mechanisms behind its self-assembly and the interactions between its domains is essential for elucidating its roles in cellular processes. FL FUS contains IDRs and structured domains such as the RRM and ZnF, which together contribute to its dynamic behavior and interactions.^1^ Investigating the interplay between these domains enhances our comprehension of FUS phase behavior and its implications in cellular organization and disease-related aggregation. Our study employed AA MD simulations to investigate the FL FUS interaction network, utilizing two different force fields with nonbonded zinc parameters. NBMs highlighted extensive contacts dominated by aromatic, arginine, and polar residues within IDRs, with minimal contributions from folded domains. These findings reinforce that FUS phase separation is primarily driven by IDRs, with contributions from all residue types. While tyrosine and arginine have well-established roles in promoting LLPS, our results highlight underappreciated contributions of other polar residues, such as glutamine, to the collective interaction network.^33,88,110^

Beyond interactions, our results illuminate the significant influence of folded domains on the overall conformational ensemble and phase behavior of FL FUS. Simulations of isolated folded domains demonstrated that the RRM domain is more stable in isolation than within the FL context, suggesting that interdomain interactions destabilize RRM. Specifically, the ff03ws model, with an unstable RRM domain, exhibited a collapsed conformational ensemble, while the same model with a RRM domain stablilzed by structural restraints had a more expanded protein conformation. This relationship between RRM stability and global conformation provides a mechanistic basis for the observed rapid aggregation of wild-type FL FUS droplets and the slower aggregation with stabilized RRM, emphasizing the critical role of RRM stability in modulating FUS dynamics. Similarly, our simulations revealed that bonded zinc-coordination parameters are essential for maintaining the folded state of the ZnF domain. The introduction of ZAFF significantly improved ZnF domain stability and folding accuracy, providing insights into the structural preservation within FUS and guiding future modeling of the RanBP2-type ZnF family. However, the mismatches in Zn–S distances and Zn–S–C_β_ angles suggest there is still room for improvement in the current ZAFF. These results suggest that FUS folded domains act as structural modulators, indirectly modulating phase behavior by constraining IDR dynamics rather than engaging directly in multivalent contacts.

Utilizing the modified force field (ff99SBws-STQ-ZBM), our simulation of the condensed phase revealed interaction profiles that were remarkably consistent with those observed in single-chain ensembles. Despite the expected chain expansion and increased intermolecular contacts within the condensate, structured domains maintained their native folds while exhibiting limited contributions to the overall intermolecular interaction network. This conservation of interaction patterns between dilute and condensed phases aligns with previous observations in IDP systems,^33,60–62^ but extends these principles to multidomain proteins, demonstrating that folded domains can function as passive structural scaffolds rather than active participants in the multivalent interaction network driving phase separation. Quantitative contact analysis further revealed a nuanced interplay between interaction strength and residue abundance. While certain residues exhibit strong per-residue interactions, their overall contribution to LLPS may be limited by low abundance. Conversely, prevalent residues like glycine, despite forming weaker individual contacts, can significantly impact LLPS through their high frequency. This dual dependency framework, where less frequent residues with strong contributions coexist with high-abundance residues exerting substantial collective effects through numerous weak contacts, provides critical insight into the molecular determinants of phase separation in multidomain proteins. Notably, we identified FR π-π interactions—previously hypothesized but unverified—through sp^2^/π plane angle-distance distribution analysis.^4,33,88,105,107,111^ This multimodal interaction landscape illustrates how multidomain proteins leverage diverse physicochemical forces to fine-tune phase boundaries in complex systems.

In conclusion, our simulations provide detailed insights into the molecular mechanisms of FUS LLPS and its modulation by folded domain stability. While our modified force field successfully captured key aspects of FUS behavior—including folded domain stability and IDR interaction patterns—persistent discrepancies in global chain dimensions (*e.g.*, *R*_g_ and *R*_h_) relative to experimental data highlight unresolved challenges in force field parametrization. These limitations likely stem from an imbalance in the strength of protein-water interactions and residue-specific inaccuracies for torsional potentials governing disordered regions.^57,58,112^ Future studies could address these gaps by integrating machine learning-derived corrections or experimental constraints from techniques like SAXS or single-molecule FRET.^113^ Importantly, our findings bridge a critical gap in the LLPS field by uncovering the interplay between folded and disordered regions in multidomain proteins. By demonstrating that single-chain simulations recapitulate condensate interaction profiles, we establish a computationally tractable framework for studying phase separation in other complex systems. These insights not only advance our mechanistic understanding of FUS-driven cellular organization but also provide a structural blueprint for designing therapeutics targeting specific interactions—such as small molecules that stabilize RRM domains to inhibit pathological aggregation in ALS and FTD.

## Supplementary Material



## Notes

Summary of the performed single-chain all-atom simulations (Table S1); system size of the full-length fus all-atom slab simulation (Table S2); dynamic conformational ensemble in atomistic simulations (Figure S1); stability of folded domains (Figure S2); zinc-binding within the ZnF domain (Figure S3); cnformations between folded domains and full-length constructs (Figure S4); residue pair contacts from FL FUS single-chain simulations (Figure S5); chain dimensions within a FL FUS condensate (Figure S6); FL FUS single-chain ensemble guides condensate formation (Figure S7) (PDF)
