# Supplementary material for: Elucidation of the Molecular Interaction Network Underlying Full-Length FUS Conformational Transitions and Its Phase Separation Using Atomistic Simulations
Source: J Phys Chem B. 2025 Aug 22;129(35):8843–57. doi: 10.1021/acs.jpcb.5c02911 (PMC12415842; doi:10.1021/acs.jpcb.5c02911)
Supplement: Supplementary file 1 [file jp5c02911_si_001.pdf]

Supporting Information

Elucidation of the molecular interaction network underlying full-length FUS conformational transitions and its phase separation using atomistic simulations

Shuo-Lin Weng[1,*], Priyesh Mohanty[2], Jeetain Mittal[1,2,3,*]

[1]Department of Chemistry, Texas A&M University, College Station, TX 77843

[2]Artie McFerrin Department of Chemical Engineering, Texas A&M University, College Station, TX 77843

[3]Interdisciplinary Graduate Program in Genetics and Genomics, Texas A&M University, College Station, TX 77843

*Correspondence: Shuo-Lin Weng - slweng@tamu.edu, Jeetain Mittal - jeetain@tamu.edu

Force field	Detail	Protein	MD engine	Simulation time
03ws	ff03ws + CYZ	FL FUS	Amber	10 μs * 3 replicas
		RRM	Amber	5 μs * 3 replicas
		ZnF	Amber	5 μs * 3 replicas
99SBws-STQ	ff99SBws-STQ + CYZ	FL FUS	Amber	5 μs * 3 replicas
		RRM	Amber	5 μs * 3 replicas
		ZnF	Amber	5 μs * 3 replicas
03ws RRM restraint	ff03ws + CYZ + RRM restraints	FL FUS	OpenMM	5 μs * 3 replicas
03ws-ZBM	ff03ws + ZAFF	FL FUS	OpenMM	5 μs * 3 replicas
		ZnF	Amber	5 μs * 1 replicas
99SBws-STQ-ZBM	ff99SBws-STQ + ZAFF	FL FUS	OpenMM	5 μs * 3 replicas
		ZnF	Amber	5 μs * 1 replicas
03ws-ZBM RRM restraint	ff03ws + ZAFF + RRM restraints	FL FUS	OpenMM	5 μs * 3 replicas

Table S1. Summary of the Performed Single-Chain All-Atom Simulations

Force field	ff99SBws-STQ + ZAFF, TIP4P/2005
Number of chains	25
Number of amino acids per chain	526
Number of water molecules	196161
Number of Zn^{2+}	25
Number of Na^{+}	556
Number of Cl^{-}	856
Total number of atoms	963231
Equilibrated box dimension (nm)	12.3 x 12.3 x 49.0
Ionic strength (mM)	158
Amber 22 benchmark on A100 GPU	52 ns / machine day
Amber 22 production	2.5 μs NVT

Table S2. System Size of the Full-length FUS All-Atom Slab Simulation

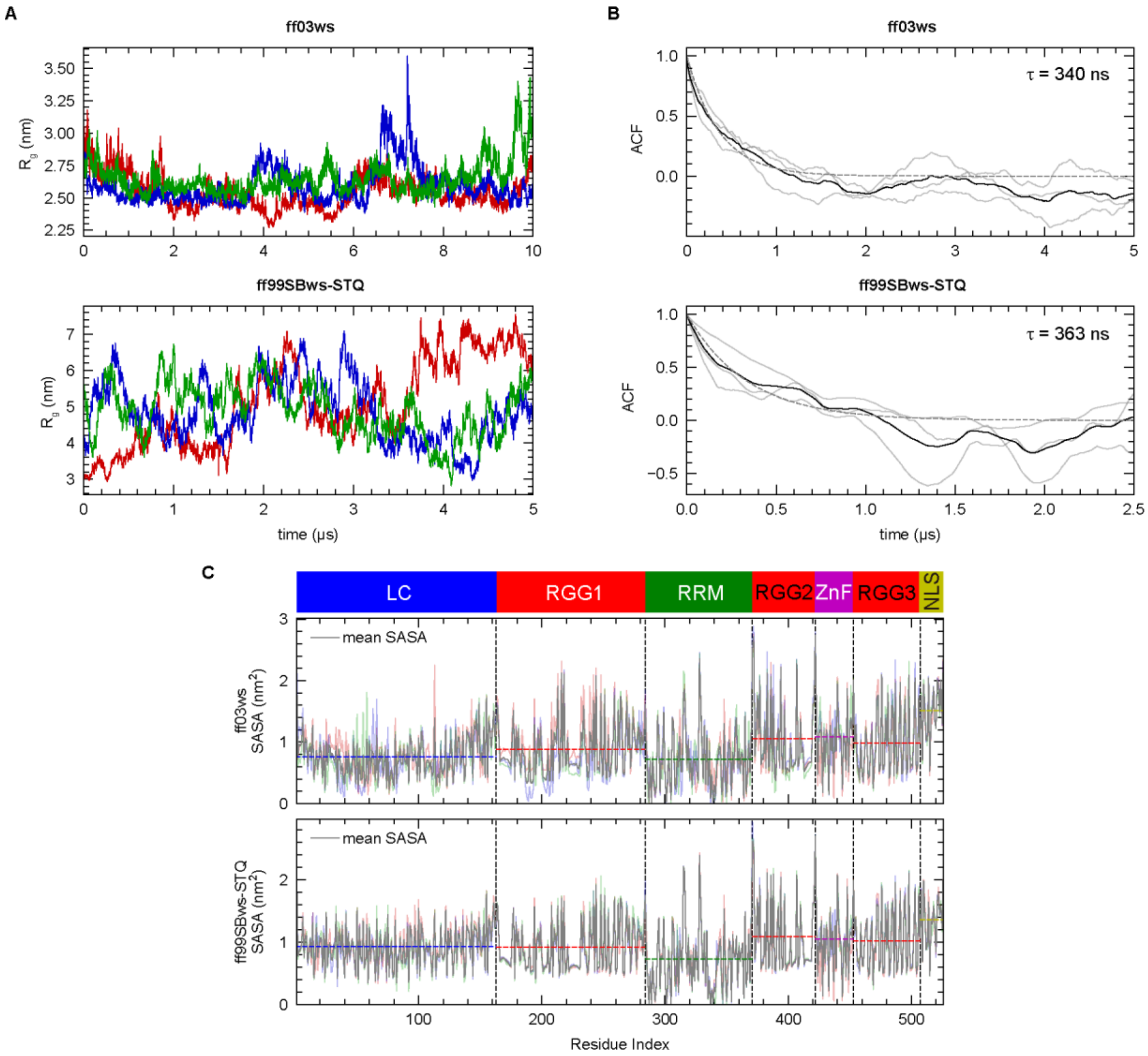

Figure S1. Dynamic conformational ensemble in atomistic simulations. A. R_g of FL FUS as a function of time from three independent replicas using two force fields with nonbonding parameters. **B.** R_g autocorrelation function (ACF) for three independent replicas (grey) and mean correlation function (black). Conformational relaxation time is estimated by fitting the mean C(t) profile to a single exponential function (dashed black line). **C.** Per-residue Solvent Accessible Surface Area (SASA) analysis on the FL FUS single-chain simulations. Colored dashed lines represent the mean per-residue SASA values of each domain.

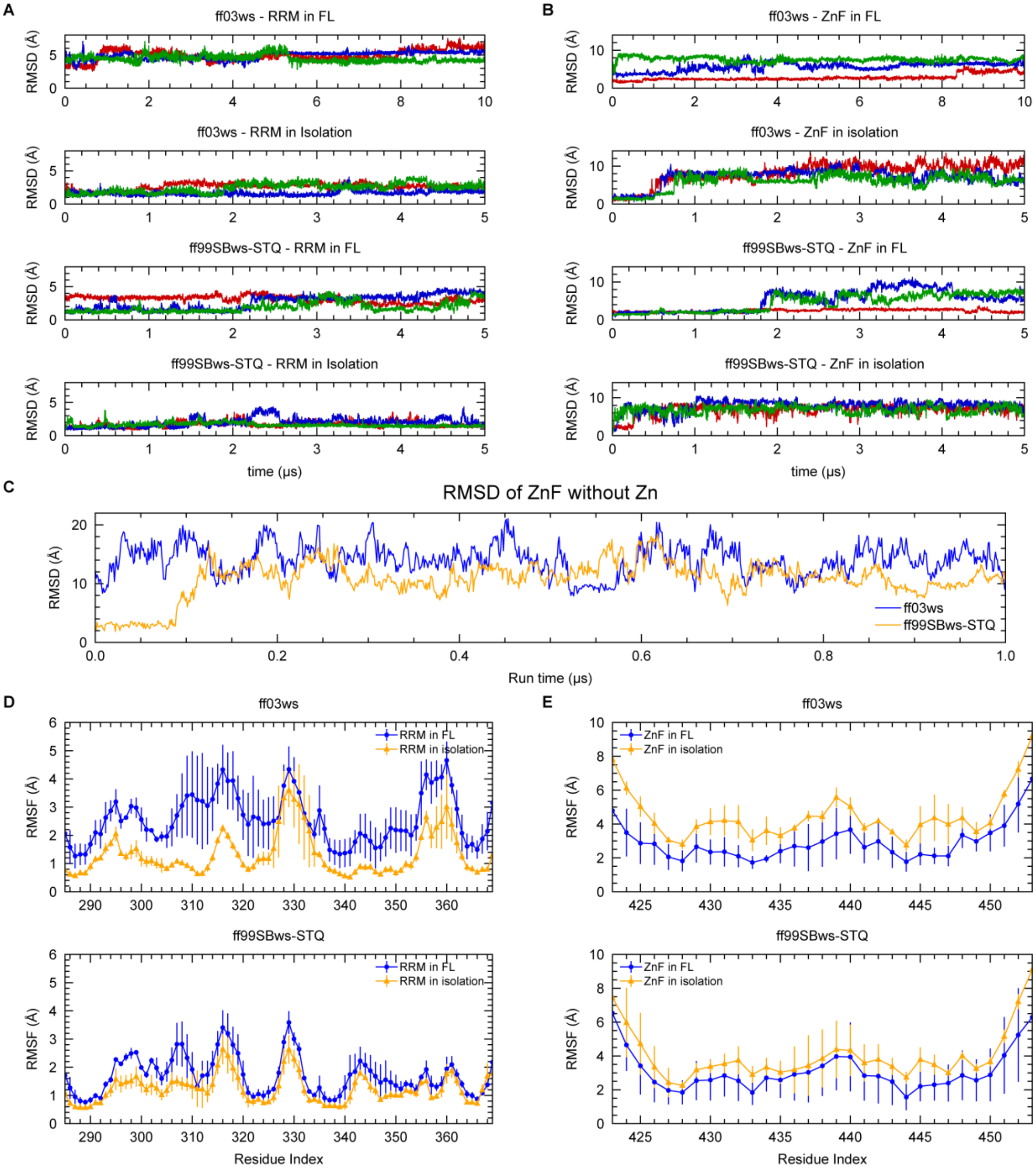

Figure S2. Stability of folded domains. A. B. Cα RMSD of the RRM and ZnF domains as a function of time across three independent replicas for each condition. **C.** Cα RMSD of the ZnF domain over time from isolated ZnF simulations without the zinc ion. **D. E.** Root-mean-square fluctuation (RMSF) of Cα atoms in the RRM and ZnF domains.

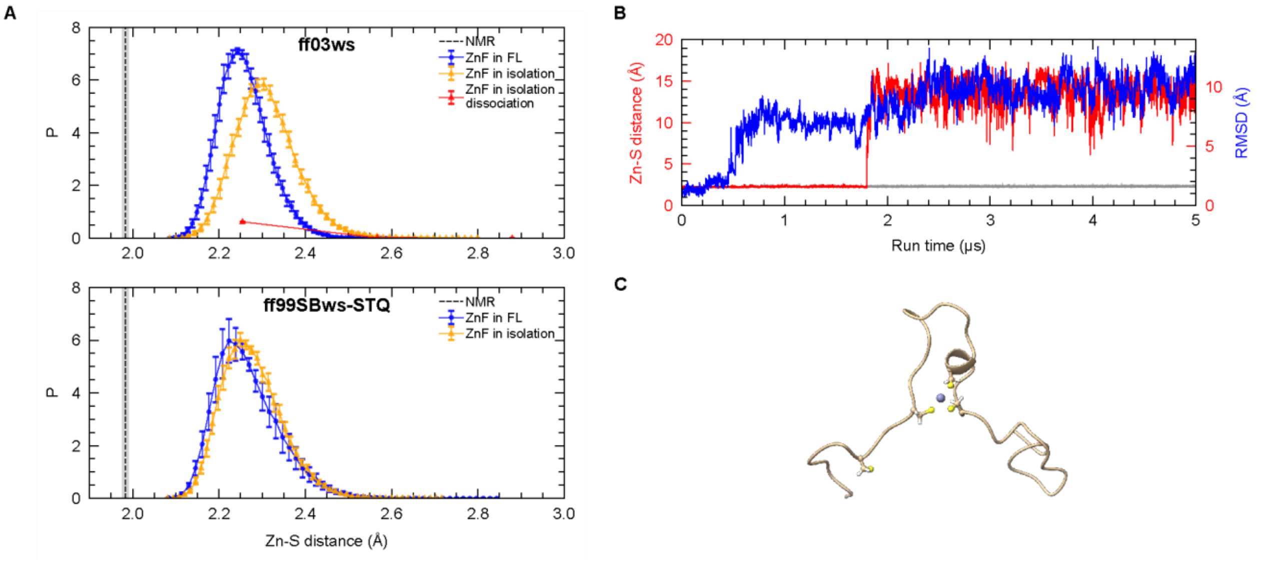

Figure S3. Zinc-binding within the ZnF domain. A. Distributions of Zn–S distances across all four cysteines for each condition from three replicas, compared to the NMR ensemble. One isolated ZnF simulation with ff03ws showed cysteine detachment. **B.** Zn–S distance (red) and RMSD (blue) over time for the detached cysteine instance. **C.** Snapshot of the detached ZnF structure. Cysteine residues and zinc were shown.

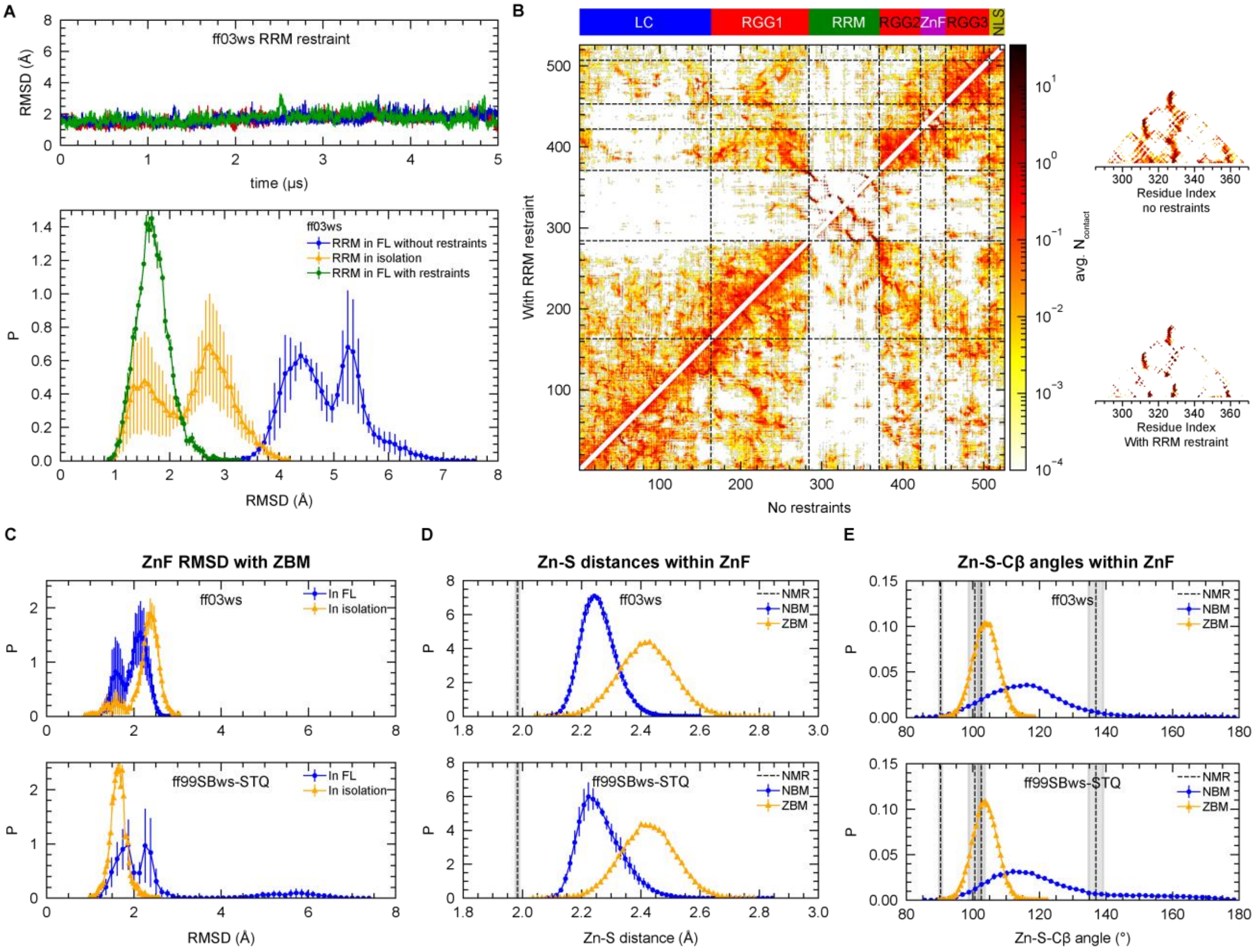

Figure S4. Conformations between folded domains and full-length constructs. A. Top: Cα RMSD of the RRM domain over time from three independent replicas of FL FUS simulations with ff03ws and RRM restraint. Bottom: Cα RMSD distributions of the RRM domain with ff03ws for FL FUS with and without RRM restraint, and for the isolated RRM without restraints. **B.** Intramolecular contact profiles from FL FUS single-chain simulations using ff03ws, with and without RRM restraints. Contact profiles of RRM are also shown. **C.** Cα RMSD distributions of the ZnF domain using ZBM parameters, comparing FL FUS to the isolated ZnF domain. **D.** Distributions of Zn–S distances in the ZnF domain of FL FUS simulations using NBM or ZBM parameters, across three replicas and compared to the NMR ensemble. **E.** Distributions of Zn–S–Cβ angles in the ZnF domain of FL FUS simulations using NBM or ZBM parameters, across three replicas and compared to the NMR ensemble.

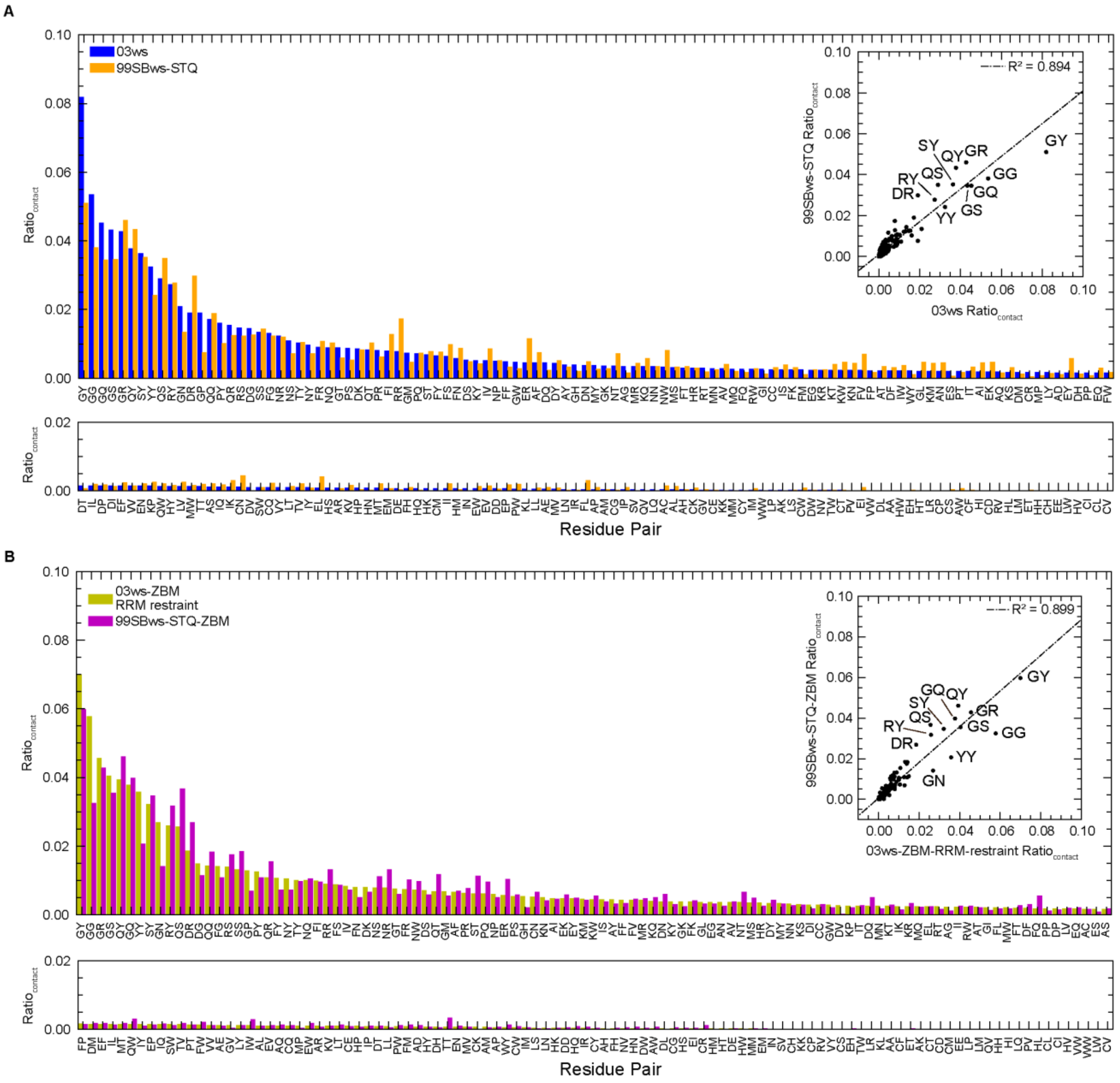

Figure S5. Residue pair contact ratios from FL FUS single-chain simulations. A. With unmodified force fields. Inset: Correlation between pairwise contact ratios in the ff03ws (x-axis) and ff99SBws-STQ (y-axis) models. **B.** With modified force fields. Inset: Correlation between pairwise contact ratios in the ff03ws-ZBM RRM restraint (x-axis) and ff99SBws-STQ-ZBM (y-axis) models. Same as **Figure 5** Inset.

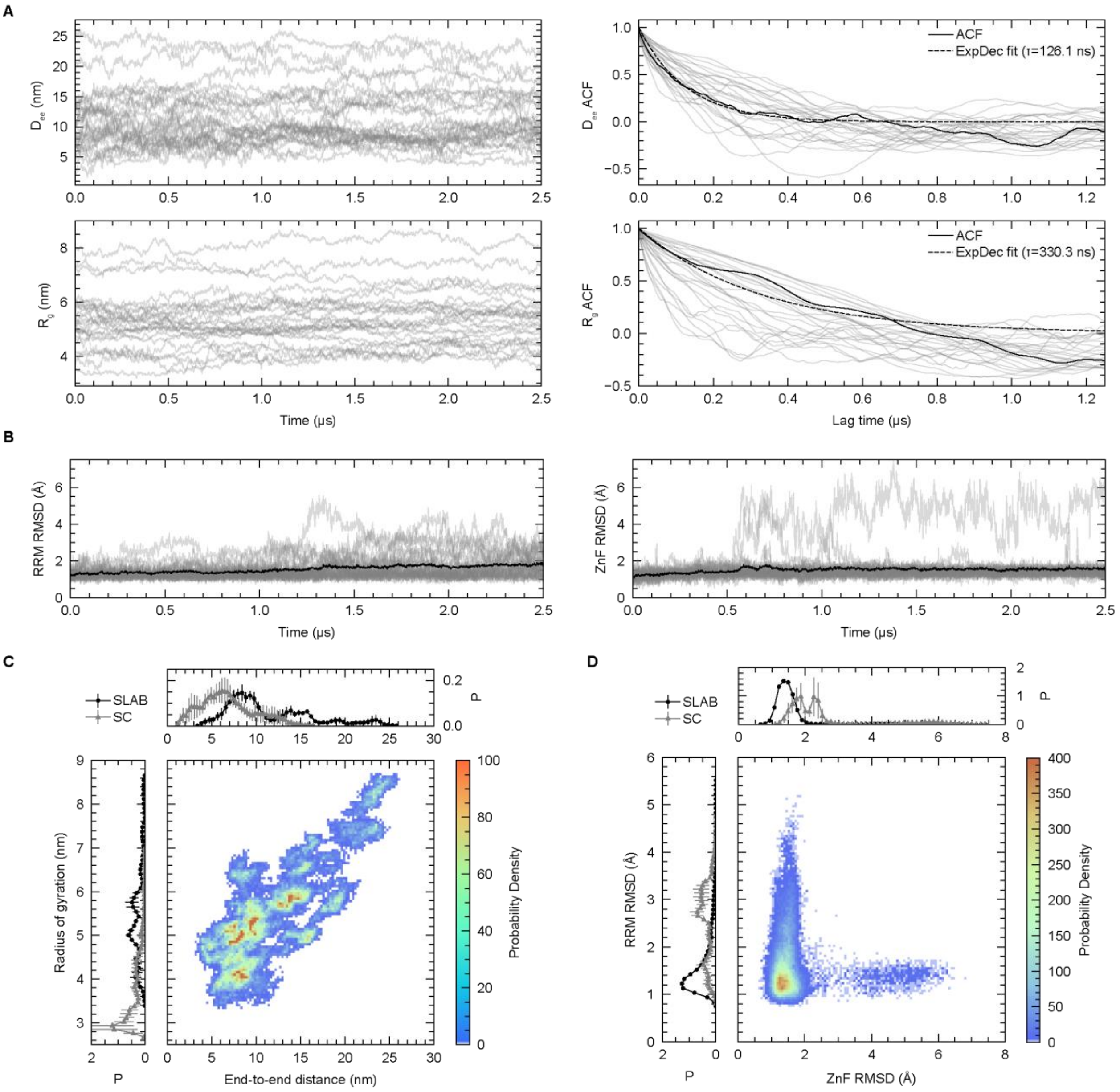

Figure S6. Chain dimensions within a FL FUS condensate. A. End to end distance (D_{ee}) and radius of gyration (R_g) as a function of time of each chain within the FL FUS condensed phase simulation, and their corresponding autocorrelation functions, for each chain (grey) and mean correlation function (black). Relaxation time is estimated by fitting the mean profile to a single exponential function (dashed black line). **B.** Cα RMSD of the RRM and ZnF domains over time for each chain (gray) and mean value (black) within the condensed phase simulation.

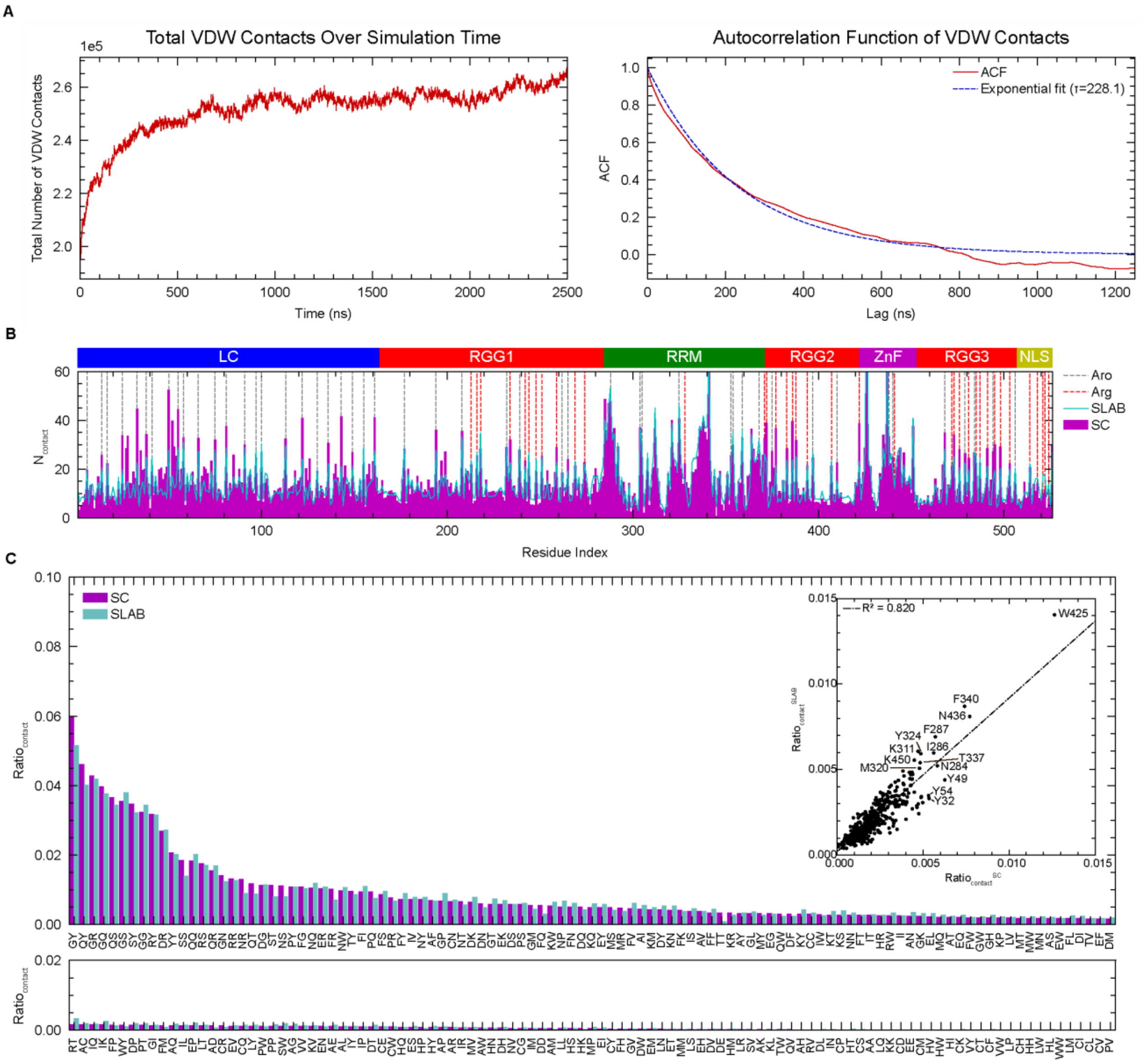

Figure S7. FL FUS single-chain ensemble guides condensate formation. A. Total number of contacts as the function of time calculated from the FL FUS condensed phase simulation and the corresponding autocorrelation. Relaxation time is estimated by fitting the mean profile to a single exponential function (dashed blue line). **B.** One-dimensional summations of contacts from SC and SLAB simulations. The positions of aromatic (Aro) and arginine (Arg) residues are indicated. **D.** Residue pair contacts from FL FUS SC and SLAB simulations. Inset: Correlation between 1D contact ratio summations (per-residue) in the SC (x-axis) and SLAB (y-axis) simulations.
